# Information and Communication Systems to Tackle Barriers to Breastfeeding: Systematic Search and Review

**DOI:** 10.2196/13947

**Published:** 2019-09-27

**Authors:** Kymeng Tang, Kathrin Gerling, Wei Chen, Luc Geurts

**Affiliations:** 1 e-Media Research Lab KU Leuven Leuven Belgium; 2 Center for Intelligent Medical Electronics Fudan University Shanghai China

**Keywords:** breastfeeding, technology, review

## Abstract

**Background:**

Breastfeeding has many benefits for newborns, mothers, and the wider society. The World Health Organization recommends mothers to feed newborns exclusively with breastmilk for the first 6 months after birth, but breastfeeding rates in many countries fail to align with the recommendations because of various barriers. Breastfeeding success is associated with a number of determinants, such as self-efficacy, intention to breastfeed, and attitudes toward breastfeeding. Information and communication technology (ICT) has been leveraged to support breastfeeding by means of improving knowledge or providing practical supports in different maternal stages. Previous reviews have examined and summarized the effectiveness and credibility of interventions; however, no review has been done from a human-computer interaction perspective that is concerned with novel interaction techniques and the perspective of end users.

**Objective:**

The objective of this review was to provide a comprehensive overview of existing digital interventions that support breastfeeding by investigating systems’ objective, technology design, validation process, and quality attributes, both in terms of clinical parameters as well as usability and user experience.

**Methods:**

A systematic search was conducted in accordance with Preferred Reporting Items for Systematic Reviews and Meta-Analyses guidelines in the following libraries: PubMed, Science Direct, Association for Computing Machinery Digital Library (ACM Digital Library), and Institute of Electrical and Electronics Engineers Xplore (IEEE Xplore).

**Results:**

A total of 35 papers discussing 30 interventions were included. The main goals of these interventions were organized into 4 categories: breastfeeding education (n=12), breastfeeding promotion (n=8), communication support (n=6), and daily practical support (n=4). Of the interventions, 13 target mothers in the postnatal period. Most interventions come in forms of client communication systems (n=18), which frequently leverage Web technologies, text message, and mobile apps to provide breastfeeding support. Systems predominantly focus on mothers; validation strategies were rather heterogeneous, with 12 user studies concerning usability and user experience and 18 clinical validation studies focusing on the effects of the interventions on breastfeeding determinants; 5 papers did not report results. Generally, straightforward systems (eg, communication tools or Web-based solutions) seem to be more effective than complex interventions (eg, games).

**Conclusions:**

Existing information and communication systems offer effective means of improving breastfeeding outcomes, but they do not address all relevant periods in parenthood (eg, the antenatal period) and often do not involve important stakeholders, such as partners. There is an opportunity to leverage more complex technical systems to open up avenues for the broader design of ICT to support breastfeeding; however, considering evaluation outcomes of existing support systems of higher complexity, such systems need to be designed with care.

## Introduction

### Background

Breastfeeding has many benefits for infants, mothers, and the wider society. A meta-analysis by Victora et al [1] suggests that infants who are breastfed show better immunity to child infections, are less likely to have oral occlusion misalignment and diabetes, and have higher intelligence [1,2]. Faster uterus recovery after delivery, shorter weight stabilization period [3], and natural contraception [1] are some of the immediate benefits for breastfeeding mothers. Other positive lifelong effects for breastfeeding mothers include protection against breast cancer and other diseases [1]. Beyond improved health outcomes, breastfeeding has far-reaching economic implications, such as a huge saving on health care costs [4,5]. However, breastfeeding rates in many countries fall short of achieving the World Health Organization (WHO) directive that recommends mothers to continue to exclusively breastfeed for 6 months [6].

Breastfeeding success is associated with a number of barriers and facilitators. Studies [7,8] have shown that birth complications, mode of delivery, medical conditions of mothers and infants, and physical availability negatively affect exclusiveness and initiation of breastfeeding. Socioeconomic parameters including age, marital status, income, education, and getting back to work also affect breastfeeding duration [7,9-11].

Self-efficacy [12], the perception of milk supply [9,13-17], and initiation and strength of intention to breastfeed [18-20] are reported as modifiable breastfeeding determinants. Self-efficacy, for example, is associated with the perception of being supported [9], exposure to breastfeeding activities [9], early breastfeeding practice [5], and past experience [9]. Perception of milk supply, on the other hand, depends on mothers’ self-efficacy and level of knowledge and skills [12]. Sufficient breastfeeding knowledge and skills may help mothers avoid physical discomfort [21]. Breastfeeding initiation and intention are influenced by subjective norms [5], acknowledgment of the benefits [5], attitudes toward breastfeeding, and perception of being supported from mothers’ social network [5,9], for example, family, partners, and health care professionals. Partners, in particular, potentially contribute to breastfeeding maintenance and feeding plan decision [22].

### Prior Works

Information and communication technology (ICT) has been leveraged for breastfeeding support, for example, to provide breastfeeding education [23], through persuasive systems designed to encourage breastfeeding [24] or provide advice throughout the process [25]. Existing review papers [26-29] and meta-analyses [30] have addressed the credibility and effectiveness of specific technology-based interventions (eg, phone calls [28], websites [26], or mobile app use in China [27]). However, there is no comprehensive analysis of digital solutions to support breastfeeding from the perspective of technology design, taking into account the end users who systems are designed for, what experience they provide for the end users, and how they relate to the barriers and facilitators of breastfeeding. Here, we address this issue to map the landscape of the currently available solutions to support breastfeeding and identify challenges and opportunities for future studies in this area.

### Objectives

This review aims to give an overview of the currently available digital interventions to address barriers and facilitators to breastfeeding by investigating the trend in technology design through the lens of human-computer interaction that focuses on the design, development, and evaluation of technology to solve real-world challenges that involve end users. Through this review, we reveal strengths and weaknesses in existing systems and underlying technologies, thereby identifying future opportunities for researchers in human-computer interaction, digital health, and public health, and hope to inform the work of health care professionals.

Toward these goals, we seek to explore this space via the following research questions:

*RQ1.* What type of ICT-based breastfeeding-supporting systems are available? Who are they intended for?

*RQ2.* How did the systems integrate into health care provisions to support breastfeeding? What technology platforms were used to achieve their goals?

*RQ3.* Were the existing systems validated in terms of the experience they provide for users and their effectiveness in clinical terms?

## Methods

### Search Criteria and Procedure

**Figure 1:**
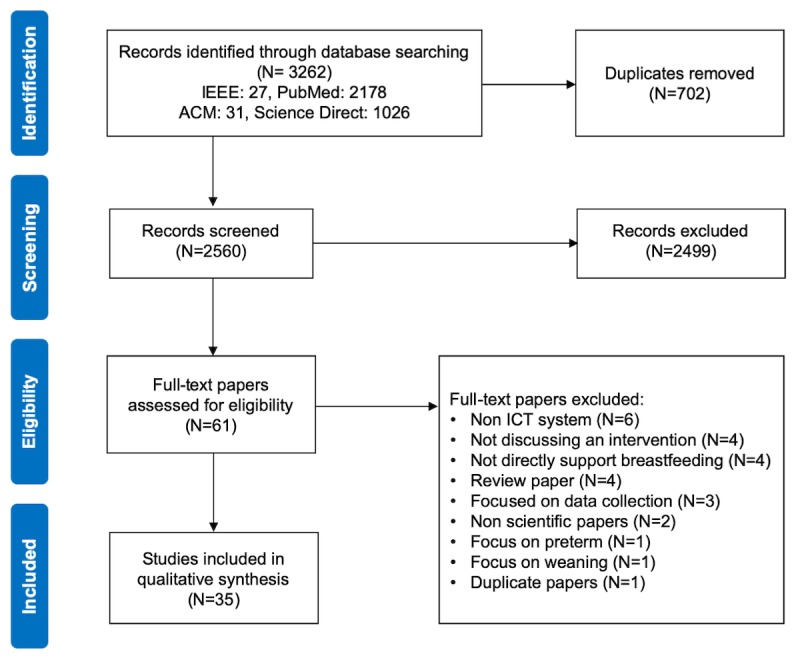
Preferred Reporting Items for Systematic Reviews and Meta-Analyses (PRISMA) flowchart of the paper selection process. IEEE: Institute of Electrical and Electronics Engineers; ACM: Association for Computing Machinery; ICT: information and communication technology.

This review follows the Preferred Reporting Items for Systematic Reviews and Meta-Analyses [31] guidelines (see Figure 1) to select and process papers. Reflecting on our goals that entail a survey of interventions across disciplines, we queried papers in technical and medical libraries: Institute of Electrical and Electronics Engineers Xplore (IEEE Xplore), Association for Computing Machinery Digital Library (ACM Digital Library), Science Direct, and PubMed. We searched for papers that mentioned the terms: *breastfeeding* and *technology* and their variations in Title, Abstract, and Keywords fields. Combinations of search terms in the search query were modified for every database to preserve the intent of the query.

### Paper Selection Process

We queried papers published before November 2018 using a search strategy specified in Table 1. A total of 3262 resulting items were imported to Elsevier’s Mendeley Desktop 1.9 for duplicates removal. At this stage, 702 duplicates were removed. On the basis of predefined inclusion and exclusion criteria, we performed a 2-phase eligibility scan: (1) title and abstract screening and (2) full-text screening for eligibility.

#### Inclusion Criteria

We included papers discussing digital interventions that satisfied all the following criteria: (1) full text in English, (2) targeted healthy human subjects regardless of breastfeeding role, and (3) provided direct or partial breastfeeding support.

We also included publications that discussed a system from different perspectives, for example, from experience-centric and clinical aspects. Work-in-progress papers were also included if their fully implemented system was not available.

#### Exclusion Criteria

We excluded papers that met any of the following criteria: (1) contained only abstract, (2) targeted subjects with special medical conditions, for example, preterm infants or severely sick mothers, and (3) reviews, books, book chapters, or reports or papers from scientific magazines.

If a paper of a fully implemented system is available, the work-in-progress papers of that system will be excluded.

**Table 1:** Literature search.

Terms	Search string
Breastfeeding	“breastfeeding” OR “breastfeed” OR “infant feeding” OR “bottle feeding”
	AND
Technology	“technology” OR “app” OR “application” OR “e-technology” OR “electronic health” OR “e-health” OR “ehealth” OR “mobile” OR “mobile health” OR “mhealth” OR “m-health” OR “computer” OR “internet” OR “web” OR “game” OR “play”

In the full-text assessment phase, we eliminated 26 publications that did not fit our criteria (see Figure 1). Finally, we included 35 eligible papers that described 30 digital interventions. Of the papers published, 31 were in medical or health informatics journals. The other 4 papers were presented in human-computer interaction and computer science conferences.

### Data Extraction and Analysis

As a preprocessing step, papers that discussed the same systems were grouped. We defined an extraction scheme that comprised 3 corresponding perspectives.

We first coded the goals and methods of breastfeeding support of each system to extract necessary data to answer *RQ1*. Here, systems with similar goals were grouped to craft categories from the data. For systems that fell into multiple categories, we coded the highest priority goal. We then extracted excerpts of breastfeeding-supporting methods for each system. Finally, we coded each system by its intended audience and context of use, if made explicit by the authors.

To identify the technology platforms used by the systems (*RQ2*), we coded the technology used in the systems and the motivation behind the choice of platform for each system. In this step, we classify the systems according to the system classification framework for digital health intervention 1.0 of WHO [32]. After that, we further classify the systems by their technology platforms and rationale behind the choice of technology.

The last data extraction step is to identify the validation process and the reported effectiveness of the systems (*RQ3*). The papers were first examined to identify whether a user study or a clinical study had been conducted; a paper may be coded twice if it discusses both types of studies. In case of user studies, we coded their methods and results (eg, controlled lab study or in-the-wild deployment). For clinical studies, we extracted outcome measures, the statistical significance of results, the number of participants in the study, and possible limitations of results, if explicitly stated.

## Results

Here, we present our results in line with the research questions.

### Goals of Information and Communication Systems to Support Breastfeeding and Target Audience

This section aims to address *RQ1* by showing an overview of the identified interventions (summarized in Table 2) supplemented by a summary of their intended users and the context of use in Table 3. The interventions were organized based on their main objectives and the methods of breastfeeding support. Here, 4 main clusters emerged: (1) breastfeeding education for mothers and training tools for health care professionals, (2) breastfeeding promotion using persuasive techniques, (3) communication tools for mothers, partners, and health care professionals, and (4) daily practical breastfeeding support.

#### Breastfeeding Education

In total, 11 of the 30 systems aimed to provide breastfeeding education. Here, we separate educational interventions for mothers and training tools for health care practitioners.

A total of 9 papers [25,33-40] studied 8 educational interventions for mothers. The majority of these interventions focused on digitizing existing knowledge into generic learning modules with multimedia to aid explanation [25,34,35,38-40]. Geoghegan–Morphet et al [25] offered a Web-based forum in addition to educational resources. Besides these generic learning programs, some educational interventions were tailored. For instance, Abbass et al [33] involved an indigenous community to craft culturally relevant educational resources. Similarly, Joshi et al [35,39] customized educational content to suit Hispanic mothers.

Other modified generic systems for mothers emphasized learning via interactive exploration. For example, an interactive agent [36] guided mothers to explore different aspects of the breastfeeding process through simulated conversations. Grassley et al [37] proposed a quest-based game for mothers to build up their breastfeeding knowledge by completing playful quests. The game engaged users in various Web-based learning activities, such as reading and watching breastfeeding-related multimedia contents.

We identified 6 papers [23,41,42,44-46] that discussed 4 training tools for health care professionals. These systems aimed to improve breastfeeding knowledge and support skills. A total of 3 papers [42,45,46] evaluated Breastfeeding Basics [43], a Web intervention that provided modular breastfeeding educational resources and training materials for health care practitioners. A total of 2 papers [23,41] focused on designing course contents to be published on off-the-shelf e-learning platforms, such as Moodle and Blackboard Learn. Meanwhile, 1 study [44] made use of a website with 2 separated forums: one for pediatric residents and the other for breastfeeding mothers. This intervention allowed the pediatric residents to learn from their peers on one forum and apply their new skills when supporting mothers on the other forum.

**Table:** Summary of the included papers.

Reference		Intervention description	Intended user	Usage context
**Breastfeeding education for mothers and training tools for health care professionals**				
	Abbass–Dick et al, 2018 [33]	Breastfeeding Web resources for indigenous audiences	Mothers	Unspecified
	Cheng et al, 2003 [34]	General breastfeeding Web resources	Parents and parents-to-be	Prenatal
	Joshi et al, 2015 [35]	Bilingual breastfeeding education on touch screen kiosks	Mothers	Postnatal
	Edwards et al, 2013 [36]	Breastfeeding consultation with a computer agent	Mothers	Pre- and postnatal
	Grassley et al, 2017 [37]	Breastfeeding quest game	Mothers	Prenatal
	Huang et al, 2007 [38]	Web-based breastfeeding education program	Mothers	Prenatal
	Joshi et al, 2016 [39]	Bilingual breastfeeding education on touch screen kiosks	Mothers	Postnatal
	Labarere et al, 2011 [40]	Computer-based breastfeeding lessons on CD-ROM	Mothers	Postnatal
	Geoghegan–Morphet et al, 2014 [25]	Web-based breastfeeding resource and virtual infant feeding support clinic	Parents and parents-to-be	Postnatal
	Cianelli et al, 2015 [23]	Breastfeeding electronic learning program on Blackboard Learn platform	Caregivers	Nursing school
	Colaceci et al, 2017 [41]	Breastfeeding electronic learning program on Moodle platform	Caregivers	Career training
	Deloian et al, 2015 [42]	Publicly available breastfeeding Web education—Breastfeeding Basics [43]	Caregivers	Unspecified
	Lasarte Velillas et al, 2007 [44]	Breastfeeding education via Web forums	Mothers and caregivers	Postnatal
	Lewin and O’Connor, 2012 [45]	Publicly available breastfeeding Web education—Breastfeeding Basics [43]	Caregivers	Unspecified
	O'Connor et al, 2011 [46]	Publicly available breastfeeding Web education—Breastfeeding Basics [43]	Caregivers	Unspecified
**Breastfeeding encouragement**				
	Wardle et al, 2018 [47]	Milk Matters app to facilitate breastmilk donation	Mothers	Postnatal
	Gallegos et al, 2014 [48]	Weekly 2-way SMS^a^ to tackle breastfeeding challenges and encourage positive feeding practices	Mothers	Postnatal
	Hmone et al, 2017 [24]	Breastfeeding promotion via SMS	Mothers	Pre- and postnatal
	Jiang et al, 2014 [49]	SMS to improve breastfeeding practice	Mothers	Pre- and postnatal
	Litterbach et al, 2017 [50]	Multichannel infant feeding support and motivation	Mothers	Postnatal
	Maslowsky et al, 2016 [51]	Breastfeeding education and support through mobile phone calls	Mothers	Postnatal
	Zunza et al, 2017 [52]	2-way SMS and motivational interview to promote breastfeeding among HIV positive mothers	Mothers	Postnatal
	Unger et al, 2018 [53]	SMS to improve breastfeeding practices and contraception use	Mothers	Pre- and postnatal
**Communication tools**				
	White et al, 2018 [54]	Milk Man app to engage fathers to support breastfeeding mothers	Fathers	Unspecified
	Rojjanasrirat et al, 2012 [55]	Breastfeeding support via video conference	Mothers	Postnatal
	Friesen et al, 2015 [56]	Breastfeeding support via video conference	Mothers	Pre- and postnatal
	Demirci et al, 2018 [57]	Breastfeeding support via video conference on mobile	Mothers	Postnatal
	Thomas and Shaikh, 2012 [58]	Breastfeeding support over the internet (email, phone call, and Web search)	Mothers	Postnatal
	Giglia et al, 2015 [59]	Web-based resource and multichannel breastfeeding support	Mothers	Postnatal
	Ahmed et al, 2016 [60]	Web-based breastfeeding diary	Mothers	Postnatal
	White et al, 2016 [61]	Milk Man app to engage fathers to support breastfeeding mothers	Fathers	Unspecified
**Daily practical supports**				
	Balaam et al, 2015 [62]	FeedFinder app to facilitate breastfeeding location search	Mothers	Postnatal
	Wang et al, 2018 [63]	MoomMae app, breastfeeding diary and breastfeeding place finder	Mothers	Postnatal
	dela Cruz and Mendoza, 2017 [64]	MilkTrack app to facilitate breastmilk donation process	Mothers	Unspecified
	Chaovalit and Pongnumkul, 2017 [65]	MoomMae app, breastfeeding diary and breastfeeding place finder	Mothers	Postnatal

^a^SMS: short message service.

**Table 3:** Target population of the systems and context of use.

Target users and context		Reference
**Mothers**		
	Unspecified	[33,64]
	Prenatal	[37,38]
	Pre- and postnatal	[24,36,49,53,56]
	Postnatal	[35,39,44,47,48,51,52,55,57-60,62,63,65]
**Fathers**		
	Unspecified	[54,61]
**Parents and parents-to-be**		
	Prenatal	[34]
	Postnatal	[25]
**Mothers and practitioners**		
	Unspecified	[44]
**Health care professionals**		
	Unspecified	[42,45,46]
	Nursing school	[23]
	Career training	[41]

#### Breastfeeding Promotion

In the second cluster, we identified 8 publications [24,47-53] that described systems that encouraged breastfeeding. The majority [24,48,49,52,53] of the systems utilized text messages to send out personalized breastfeeding tips and encouragement messages and sample breastfeeding experiences via short text message (SMS) responses. The content of the text messages was based on focus group discussions [53], WHO guidelines, expert inputs, and literature studies [49]. A total of 2 interventions [48,52] did not specify the source of message content and 1 system [24] used the Health Belief Model [66] to frame promotion strategies. Maslowsky et al [51] proposed a two-fold intervention that required nurses to call mothers 48 hours after hospital discharge. The idea is to deliver a maternal education session and a follow-up phone call to sample experience and provide support if needed. To keep users engaged with the intervention, Wardle et al [47] encouraged mothers to donate breastmilk using positive reinforcement techniques in their mobile app. The Growing Healthy Program [50] encouraged breastfeeding with weekly personalized motivational messages and on-demand breastfeeding resources aimed at parents with low socioeconomic status.

#### Communication Support

The third cluster comprises 7 papers [54-59,61] discussing 6 interventions that facilitate communication between peers and professionals. Of the systems, 3 [55-57] provided tele-lactation consultations via video conference for mothers with limited access to maternal care. Giglia et al [59] designed a multichannel support intervention through focus group; the intervention provides lactation supports through Web-based resources, email, and video call, all at the same time. For mothers who were not satisfied with their current caregivers, Thomas and Shaikh [58] described how mothers sought breastfeeding supports on the Web, using various information and communication tools. Besides interventions for mothers, White et al [54,61,67] used the social cognitive theory to design a mobile app that fostered peer support among expecting and new fathers by engaging them in discussions. This mobile app grouped fathers based on their baby’s age and maternal stage of his partner.

#### Daily and Practical Support

In the fourth cluster, we found 3 mobile apps and a Web intervention that practically supported breastfeeding. FeedFinder [62], MoomMae [63,65], and MilkTrack [64] are mobile apps that allow mothers to find, rate, and review suitable breastfeeding locations in public spaces. Besides breastfeeding location crowdsourcing, other features were incorporated as well, for example, MilkTrack [64] provides practical breastfeeding resources and a milk donation platform, whereas MoomMae [63,65] provides a personal breastfeeding diary. Ahmed et al [60], in contrary, only provided a Web breastfeeding diary. The diary allows health care practitioners to gain insights into mothers’ breastfeeding experience and personalize their support.

On a general level, this shows that ICT to support breastfeeding addresses a broad range of goals; however, the strongest categories that emerged throughout our analysis were systems to provide breastfeeding education and promote breastfeeding.

### Technical Platforms

**Figure 2:**
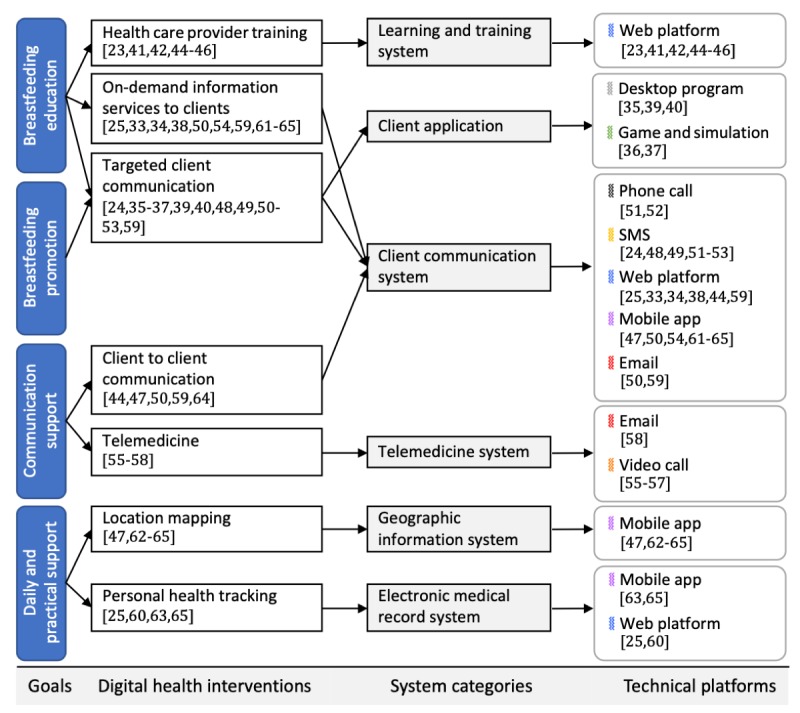
Taxonomy of the identified systems regarding intervention purpose and technology. SMS: standard message service.

To answer the second research question (*RQ2*), we characterized the digital interventions by their technology platform and system purpose. We structured our findings around the WHO system classification framework for digital health interventions [32] to maintain a mutual taxonomy across digital health domains. Here, the framework acts as connective tissues to relate system objectives to the used technology platform as shown in Figure 2. On the basis of the categorization scheme, the systems fall into the following 6 overlapping categories.

#### Client Communication Systems

Client communication systems were used in 18 digital interventions [24,25,33,34,38,44,47-54,59,61-65] to communicate breastfeeding information to targeted clients (parents), provide on-demand information services to clients, and facilitate client-to-client communication. These systems leveraged 5 technology platforms to reach their intended audiences.

*SMS* appeared in 6 interventions [24,48,49,51-53] that aimed to deliver breastfeeding information and encouraging messages to mothers. Most of them [24,48,49,52,53] were inspired by the trend of leveraging SMSs to promote healthy behavior. Affordability and high availability also contributed to the popularity of this technology [48,49,52], particularly in developing countries, as 5 SMS-based breastfeeding interventions [24,49,51-53] were studied in Myanmar, China, Ecuador, and African countries, whereas only 1 [48] was introduced in Australia. The degree of user interactions within these SMS-based interventions is rather limited: 2 systems [24,48] required users to reply with a predefined code or a syntax, whereas the other 4 [49,51-53] invited free text response, but manual human processing was required to extract the content.

*Phone calls,* which operate on the same infrastructure as SMS, were used together with SMS in 2 interventions [51,52] to compensate for the lack of in-depth communication.

A total of 2 interventions [50,59] used *email* to initiate conversations with peers [59] and deliver notifications and weekly messages in a mobile health intervention [50] that also provided personalized infant-feeding information through a mobile app.

A total of 6 *mobile apps* [47,50,54,61-65] provide on-demand information service and client-to-client communication via platforms for online discussion and information sharing. Mobile app development gained popularity among intervention designers following the ubiquity of smartphones [47,50,54,61-65]. Mobile apps can be used to establish social connectivity for parents to connect to peers and foster information sharing, for instance, review public breastfeeding spaces [62,63,65], facilitate milk donation logistic [64], and share parenting experiences [50,54,61]. In addition to social connectivity, breastfeeding information can also be provided through mobile apps [47,50,64].

A total of 6 *Web-based interventions* [25,33,34,38,44,59] were designed to provide on-demand information services and facilitate client-to-client communication. Web technologies are ubiquitous, flexible, and easy to deploy and have low operational costs [33,34,38,41,46]. They can offer a rich user-interaction through the intuitive presentation of information (eg, using multimedia or interactive content) [34,38]. On top of that, they can be tailor-made to fit the exact requirements of an intervention [33,34,38,50,60]. Intervention designers can use the Web as a supporting channel for parents to look up breastfeeding information, for example, a Web-based virtual maternity clinic [25], or as a Web-based discussion platform to connect mothers with care providers and peers [25,44,59].

#### Learning and Training Systems and Client Applications

In this group, 4 learning and training systems [23,41,42,44-46] and 4 client applications [35-37,39,40] were identified.

A total of 4 learning and training interventions [23,41,42,44-46] use *Web platforms* to improve breastfeeding knowledge of health care providers. In this context, Web-based systems become a potential learning platform because they can provide comprehensive sets of educational tools, such as lessons, knowledge evaluations [23,41], and Web-based discussion [44], and be adapted to the busy schedule of health care practitioners [41,46].

A total of 4 client applications [35-37,39,40] are self-service *desktop programs* to communicate breastfeeding information. These systems came in various shapes: interactive software running on touchscreen kiosks in clinics [39,68], software provided via CD-ROM [40], *interactive agent* that simulated lactation consultation [36], and breastfeeding quest *game* [37]. These experimental interventions aimed to explore the potential of interactive learning as an alternative mode of communicating breastfeeding information.

#### Geographic Information and Electronic Medical Record System

A total of 2 mobile apps [47,62,64] are categorized as geographic information systems, 2 Web interventions [25,60] are electronic medical record systems, and 1 mobile app [63,65] situates in both system categories.

FeedFinder [62] and MilkTrack [64], both of which also belong to the client communication system category, made use of positioning capability of smartphones to help mothers find and map suitable breastfeeding locations in public. In addition to breastfeeding location services, the MoomMae app [63,65] offers a breastfeeding diary function to keep track of mothers’ feeding habits, so they can accurately report feeding behaviors to health care professionals.

Both electronic medical record systems [25,60] are based on Web technologies. These interventions have an experience-sampling function that allows mothers to log their experience on a Web diary. The diary can be used as a personal record [25] or be accessed by health care providers to personalize their breastfeeding support tactics [60].

#### Telemedicine

Telemedicine came in forms of remote lactation consultation via video calls [55-57] and email communication [58]. All 3 identified video-call interventions [55-57] took place in the United States, which has a geographically dispersed population. Video calls break distance barriers for parents who live in rural areas [55,57] and facilitate the delivery of professional breastfeeding support when health care facilities are limited [56]. More importantly, the technology allows rich communication in real time (eg, allows health care practitioners to observe breastfeeding parents and demonstrate the feeding process [55]). Email was also used by mothers to initiate communication with health care professionals and inquire for breastfeeding information [58].

This illustrates the breadth of information and communication systems that are available to support breastfeeding, both in terms of audience and underlying technology. Here, it is interesting to note that interventions predominantly rely on existing communication channels, such as SMS or phone calls, or implement straightforward and well-explored technologies, such as Web portals.

### Validation Process and Effectiveness of Existing Systems

To understand the validation process of the systems (*RQ3*), we coded the papers into 2 categories: user studies (focused on usability, ie, determining whether systems can be used effectively and efficiently, and user experience, ie, studies that explore whether systems engage users through a positive overall experience) and clinical validation studies. A total of 5 papers [24,25,52,61,65] without discussion of results were excluded from this section.

#### User Studies

A total of 20 systems were evaluated in terms of usability, user satisfaction, and user experience. Usability and user experience of 12 systems were evaluated through user studies, whereas 8 clinical validations included measures of user satisfaction and discussions of usability flaws as a part of their evaluation process. Here, we organize the results of the studies according to their objectives.

Among the reported user studies, a breadth of quantitative and qualitative research methods was employed; participant samples included interaction design experts, health care experts, and prospective end users.

Usability was assessed with different methods, depending on the objectives of the evaluation, including expert reviews and user studies. The evaluation of MoomMae [63] involved interviews and surveys with 21 breastfeeding mothers who used the app for 4 weeks. Although the app was rated as useful, postexposure interviews revealed a negative usability trend; common flaws were confusing user interface elements, ease of use, and screen readability. The bilingual desktop breastfeeding education [35] was evaluated using Nielsen usability heuristics [69] by 2 usability experts, and the paper reported 91 usability flaws across 271 screens. Cheng et al [34] involved 20 participants in different places to evaluate a Web-based breastfeeding education system with graphical content in terms of user satisfaction and learning outcomes. The study shows that graphical content shortened view time, increased user satisfaction, and did not negatively affect learning.

Besides usability, factors contributing to user experience were also explored. The Milk Matters [47], Milk Man [61], and FeedFinder [62] apps, for example, involved end users to conduct formative tests throughout their design and development cycles to minimize usability issues and adapt to user preferences. Formative user studies with stakeholders were also conducted in the development of a culturally adapted Web-based intervention for indigenous mothers [33]. The evaluation of these systems stressed on other quality attributes, such as engagement, emotion, and ease of use in specific circumstances. The analysis of comments collected after an in-the-wild release of FeedFinder [62] app showed improvement of mothers’ confidence to breastfeed in public places. Engagement factors of the Milk Man app [61] included connectivity for fathers to seek and offer support, share personal experiences, and seek peer social connection. Likewise, the Growing Healthy Program [50] was found to be engaging because of high perceived usefulness, content that suits users’ parental beliefs, and— from a technical perspective—the use of push notifications.

Other quality measures concerned user satisfaction and perceived usefulness of the systems, although some of them did not report full-fledged user studies. In general, most of the interventions were perceived positively, but some unexpected usability flaws were discovered. For example, the CD-ROM desktop program [40] reported good reviews from participants; however, only 119 of the 240 participants in the intervention unit (a total of 993 mothers from both intervention and control groups combined) actually accessed the intervention. Likewise, participants in a 2-way SMS intervention [48] reported not being able to remember the predefined SMS response codes, suggesting that systems would need to be adapted for effective in-the-wild deployment.

#### Clinical Validation Studies

A total of 16 systems (18 papers, see Multimedia Appendix 1) were validated in terms of clinical outcomes. Of those, we found 13 systems that fully or partially achieved their objectives, 1 system validation led to inconclusive results (not statistically significant), and 2 could not demonstrate a measurable effect. In Multimedia Appendix 1, we present a summary of the clinically validated systems and indicate reported effects on outcome parameters. Here, we organize systems based on their reported outcome measures.

The breastfeeding learning and training systems for health care professionals were effective with statistical significance in contributing to the knowledge of health care professionals and supporting skills, although selection bias might exist in the studies. The 3 in-the-wild evaluations [42,45,46] of the Breastfeeding Basics [43] educational program over the course of 9, 10, and 12 years with respective numbers of 15374, 18522, and 19671 participants using pre- and posttest consistently reported improvement in all aspects of breastfeeding knowledge among the included participants. It is worth mentioning that most of the participants included in these 3 studies were required to take part in the intervention for education or professional purpose. The Blackboard-based system [23] and the forum-based intervention [44] that were evaluated based on pre- and posttests with 86 nursing students and 42 pediatrics (recruited based on self-selection) were found to be effective in improving breastfeeding knowledge. Selection bias is also found in an evaluation of the Moodle-based intervention [41], with 15004 participants that showed positive effects on breastfeeding attitude and support practice; only participants who completed and passed the first-round evaluations were included in the study.

Some of the client communication systems were also effective and statistically significant in improving breastfeeding practice and determinants: an assessment of the clinic touchscreen kiosk breastfeeding intervention [39] with 46 mothers indicated an improvement in knowledge, self-efficacy, and intention to breastfeeding. A Web-based breastfeeding education [38] evaluated with 65 primigravids at their 29 to 36 weeks of pregnancy succeeded in improving breastfeeding rates, knowledge, and attitudes. A total of 2 [49,53] of the 5 SMS-based systems have reported statistically significant improvement of the duration of exclusive breastfeeding at the sixth month, with study cohorts of 582 and 298 participants. The evaluation of the postnatal phone-call support [51] with 178 new mothers through a follow-up phone call 3 months after the intervention commencement has reported a higher rate of exclusive breastfeeding and lower formula feeding rate among mothers in the intervention group, when compared with the control group. From a yearlong study with 414 mothers on multichannel lactation support [59], a positive long-term effect on exclusive breastfeeding in the intervention group was reported.

Not all effective systems fully achieved their goals. Despite showing significant improvements in breastfeeding duration and exclusivity, a study of the breastfeeding diary [60] with 96 participants did not show statistical significance in a second objective, decreasing postpartum depression. Similarly, a study with 24 participants on the Growing Healthy Program [50] has indicated an improvement in mothers’ confidence in their choice of feeding method (either breastfeeding or bottle feeding) and perception of milk supply. However, feeding plans and intentions were not influenced as the decisions were taken before exposure to the intervention. A study on an SMS-based breastfeeding encouragement system [48] with 234 participants was shown effective in improving breastfeeding exclusivity but showed no statistically significant impact on self-efficacy, which might offer an explanation for its failure to increase breastfeeding rates.

Finally, client applications did not produce measurable effects. A 1-month controlled trial with a total of 993 mothers (from intervention and control groups) on a CD-ROM software [40] and a 25-participant pilot study of the breastfeeding game [37] failed to improve knowledge [37,40], breastfeeding rates [40], intention [37], and self-efficacy [37]. A pilot controlled study of the interactive agent [36] with 15 women showed no significant improvement in breastfeeding intention and self-efficacy and failed to alter attitudes toward breastfeeding. It is worth noting that these complex systems were neither custom-built to suit the end users nor involved the primary stakeholders in the development process but rather a modification of existing technology platforms.

Here, the heterogeneous nature of interventions and validation processes and outcome parameters limit the opportunity to conduct a meaningful comparison between interventions. On a general level, our results suggest that breastfeeding learning and training systems for health care professionals tend to succeed in improving breastfeeding knowledge among their intended users, whereas some client communication systems showed a positive effect on improving breastfeeding adherence. Other breastfeeding determinants were rarely influenced, and validation studies evaluating complex technology (eg, games) suggested that such systems were ineffective in their current designs.

## Discussion

### Principal Findings

This review gives an overview of digital interventions to support breastfeeding. We draw from research in computer science, engineering, and medical field to provide an overview of systems with a focus on technology, users, and outcomes. Our results show that the majority of systems were designed to address mothers in the postnatal period, either promoting or educating them about breastfeeding (*RQ1*). From a technical perspective, client communication systems were the most commonly used systems, with Web technologies, mobile apps, and SMS being the dominant platforms and only a small number of studies exploring more complex technologies, such as games (*RQ2*). System effectiveness was predominantly demonstrated in terms of improved breastfeeding knowledge, although improvement in behavioral outcomes might be because of systems that provided continuous proactive support. In terms of usability and user experience, results were mixed, with some systems failing to engage users (*RQ3*). Generally, our analysis suggests that straightforward technology fared better than complex systems, leaving room for an interesting debate. Here, we discuss these results; drawing from our findings, we further reflect on available systems through the lens of barriers and facilitators toward breastfeeding, and we outline opportunities for future research into the development of engaging technology interventions to promote breastfeeding.

### Current Trends in Information and Communication Technology to Support Breastfeeding

Our review suggests that, based on the WHO system categorization, client communication systems are the predominant group of ICT to support breastfeeding. Systems in the group use SMS, Web platforms, and mobile apps to educate, support, and encourage mothers [24,25,33,34,38,44,47-54,59,61-65]. In terms of effectiveness, results show that educational interventions [23,25,33-42,44-46] for mothers and health care professionals tend to focus on improving knowledge, self-efficacy, intention, and attitude. In contrast, systems that facilitate communication [54-59,61] and encourage breastfeeding [24,47-53] can be successful in helping mothers to maintain breastfeeding practice, possibly because of the improvement of perceived support [9]. Interestingly, more complex interventions (eg, the breastfeeding game [37]) did not lead to a significant improvement in breastfeeding knowledge. Details about the contents and technical design process of the interventions are rather limited, which make a precise conclusion about factors contributing to the success, or failure, of these systems difficult to draw; however, what is known from other studies exploring the design of games for health is that this is a complex process and requires careful consideration to produce the desired outcomes. Here, future studies should explore how more complex breastfeeding interventions can be designed in an effective manner. Finally, although educational interventions were effective in improving knowledge, very few of them improved self-efficacy, intention, and attitude. This suggests that stakeholders currently need to engage with multiple systems to obtain the full benefits of ICT in this setting. Hence, it may be beneficial to consider pathways toward holistic system development to provide digital solutions that not only improve breastfeeding knowledge but also are designed in a way that also allows them to affect other breastfeeding determinants.

### Opportunities for Future Research: Reflecting on Gaps in Existing Technology Through the Lens of Breastfeeding Determinants

When aligning the results of our analysis with breastfeeding determinants [5,7,9,12-17,21,22], it becomes clear that existing systems predominantly address knowledge and practical support, whereas other factors are omitted, particularly elements such as practical breastfeeding skills that affect the perception of milk supply and physical comfort [12]. Considering the use of ICT in this space, such a system would need to integrate more complex technology than those currently used (ie, Web-based systems and smartphone apps). Here, sensing systems offer an interesting design opportunity, and first attempts have been made to develop systems that can help track infant milk intake while breastfeeding [70,71]. Likewise, important determinants such as attitudes toward breastfeeding, intention, and initiation are not addressed by existing studies that predominantly target the postnatal period (13 of 30 systems), although the decision to breastfeed is usually taken in the third trimester of pregnancy [5,50]. Here, there would be potential for the development of technology solutions that address parents-to-be in the antenatal period, slowly introducing them to the topic, not only through educational but possibly also through experiential systems that give a glimpse at the breastfeeding process [72]. Finally, and perhaps most surprisingly, only 1 intervention [54,61] was specifically aimed at partners, despite research showing that partners in general, and fathers in particular, play a major role in a mother’s decision to breastfeed and success throughout breastfeeding journey [22]. Here, there is a large potential for future studies to be more inclusive, improving partners’ interest in breastfeeding, their knowledge, and their ability to support the breastfeeding mother. Generally speaking, future studies should explore the potential of emerging technologies, such as cross-platform interventions with multiple elements (eg, an educational app combined with channels for practical support), or immersive and tangible computing systems that can provide realistic insights into breastfeeding not just for mothers but also their partners (eg, a virtual reality breastfeeding simulation).

### Comparison With Previous Studies

To the best of our knowledge, this review is the first of its kind to offer a broad overview of the available systems by considering aspects (goals, methods of support, target audiences, usage context, technology platforms, and their rational and validation process) other than clinical effectiveness. Other systematic reviews [29,30] analyzed the effectiveness of the intervention from the medical aspect, whereas some other reviews focused on specific technologies (eg, phone calls [28], websites [26], or mobile apps [27]) rather than surveying all available systems. Unlike previous approaches, we investigated different characteristics of the interventions instead of summarizing their effectiveness (eg, via meta-analysis). Here, our intent is to inspire researchers from other disciplines (eg, computer science) to contribute to this space.

### Conclusions

There are various attempts to leverage ICT to encourage breastfeeding, aiming to improve breastfeeding education, persuade mothers to initiate and follow through with breastfeeding, and provide practical support. Our results show that although certain groups of systems are effective, they often only target 1 domain (eg, improving knowledge), requiring end users to engage with a multitude of systems to achieve good coverage. In addition, the majority of interventions exclusively targeted mothers and failed to consider other relevant stakeholders, most strikingly partners. Therefore, further study is necessary to explore how innovative concepts in ICT can be fully leveraged to provide comprehensive breastfeeding support, starting in the antenatal period and extending beyond the birth of the child, while engaging both parents. Through reflection on the way that existing systems (fail to) address determinants of breastfeeding, our review provides a first step toward outlining research opportunities for future study in this space.

## Appendix

Multimedia Appendix 1 Example of raw data retrieved from the Polar H10, Polar A370, and Tempo HR.

## Abbreviations

CD-ROM: Compact Disk Read Only Memory
ICT: information and communication technology
SMS: short message service
WHO: World Health Organization
