# Supplementary material for: Information and Communication Systems to Tackle Barriers to Breastfeeding: Systematic Search and Review
Source: J Med Internet Res. 2019 Sep 27;21(9):e13947. doi: 10.2196/13947 (PMC6818436; doi:10.2196/13947)
Supplement: Multimedia Appendix 1 [file jmir_v21i8e13947_app1.pdf]

Table A1. Summary of the effectiveness of the breastfeeding educational interventions for mothers and training tools for health care professionals that were validated clinically.

Reference	Breast feeding knowledge	Intention to breastfeed	Self-efficacy	Breast feeding rate	Breast feeding attitude	Skills to support breast feeding
Edwards et al, 2013 [36]	—	↑[a]n[b]	↑n	—	O[c]	—
Grassley et al, 2017 [37]	—	O	O	—	—	—
Huang et al, 2007 [38]	↑	—	—	↑	↑	—
Joshi et al, 2016 [39]	↑	↑	↑	—	—	—
Labarere et al, 2011 [40]	O	—	—	O	—	—
Cianelli et al, 2015 [23]	↑	—	—	—	—	—
Colaceci et al, 2017 [41]	—	—	—	—	↑	↑
Deloian et al, 2015 [42]	↑	—	—	—	—	—
Lasarte Velillas et al, 2007 [44]	↑	—	—	—	—	—
Lewin and O'Connor, 2012 [45]	↑	—	—	—	—	—
O'Connor et al, 2011 [46]	↑	—	—	—	—	—

[a]↑ indicates increase.
[b]n indicates not statistically significant.
[c]O indicates no effect.
— not applicable

Table A2. Summary of the effectiveness of the breastfeeding promotion/encouragement interventions that were validated clinically.

Reference	Intention to breastfeed	Self-efficacy	Breast feeding rate	Breast feeding duration	Breast feeding exclusivity	Confidence in feeding methods	Milk supply perception
Gallegos et al, 2014 [48]	—	↑[a]n[b]	O[c]	—	↑	—	—
Jiang et al, 2014 [49]	—	—	—	↑	↑	—	—
Litterbach et al, 2017 [50]	O	—	—	—	—	↑	↑
Maslowsky et al, 2016 [51]	—	—	—	—	↑	—	—
Unger et al, 2018 [53]	—	—	—	↑	↑	—	—

[a]↑ indicates increase.
[b]n indicates not statistically significant.
[c]O indicates no effect.
— not applicable

Table A3. Summary of the effectiveness of the communication support systems that were validated clinically.

Reference	Breast feeding duration	Breast feeding exclusivity	Postpartum depression
Giglia et al, 2015 [59]	↑[a]	—	—
Ahmed et al, 2016 [60]	↑	↑	↓[b]n[c]

[a]↑ indicates increase.
[b]↓ indicates decrease.
[c]n indicates not statistically significant.
— not applicable
